# Critical Role of Myeloid-Derived Suppressor Cells in Tumor-Induced Liver Immune Suppression through Inhibition of NKT Cell Function

**DOI:** 10.3389/fimmu.2017.00129

**Published:** 2017-02-13

**Authors:** Hongru Zhang, Zheng Li, Li Wang, Gaofei Tian, Jun Tian, Zishan Yang, Guangchao Cao, Hong Zhou, Liqing Zhao, Zhenzhou Wu, Zhinan Yin

**Affiliations:** ^1^State Key Laboratory of Medicinal Chemical Biology, College of Life Sciences, Nankai University, Tianjin, China; ^2^Shenzhou Space Biotechnology Group, Beijing, China; ^3^The First Affiliate Hospital, Biomedical Translational Research Institute, Guangdong Province Key Laboratory of Molecular Immunology and Antibody Engineering, Jinan University, Guangzhou, China; ^4^Department of Immunology, Nanjing Medical University, Nanjing, China; ^5^Collaborative Innovation Center for Biotherapy, Sichuan University, Chengdu, Sichuan, China

**Keywords:** remote tumor, MDSC, TGF-β, CXCR2, liver immune suppression

## Abstract

Metastasis followed by the tumor development is the primary cause of death for cancer patients. However, the underlying molecular mechanisms of how the growth of tumor resulted in the immune suppression, especially at the blood-enriched organ such as liver, were largely unknown. In this report, we studied the liver immune response of tumor-bearing (TB) mice using concanavalin A (Con A)-induced hepatitis model. We demonstrated that TB mice displayed an immune suppression phenotype, with attenuated alanine aminotransferase levels and liver damage upon Con A treatment. We also elucidated that large amounts of myeloid-derived suppressor cells (MDSCs) being influx into the liver in TB mice and these MDSCs were essential for liver immune suppression through both depletion and reconstitution approaches. We further determined that these MDSCs selectively suppressed the IFN-γ production deriving from NKT cells through membrane-bound transforming growth factor β (TGF-β). Finally, we defined a tumor-derived TGF-β-triggered CXCL1/2/5- and CXCR2-dependent recruitment of MDSC into the liver. In summary, our results defined a novel mechanism of liver immune suppression triggered by growing living tumor and provided possible therapeutic targets against these MDSCs.

## Introduction

Cancer continues to represent global health problems in human beings. Emerging evidence has shown that tumors can escape immune surveillance *via* producing an immune-suppressive environment in the development and progression of tumor metastasis. Tumor-induced immunosuppression has been recognized as an essential element in tumor progression (1). Research has shown that cancer cells consistently induce local immunosuppression and then create systemic immunosuppression *via* immune-suppressive cells and cytokines (2). However, the underlying molecular mechanisms are not clear. Recent reports showed that immune responses in cancer patients are negatively regulated by immunosuppressive cells, mainly T regulatory cells (Tregs) and myeloid-derived suppressor cells (MDSCs), which suppress exuberant immune system activation and promote immunologic tolerance (3, 4). Lines of studies further defined that MDSCs can modulate the *de novo* development and induction of Tregs (5). MDSCs are known to synergize with Tregs to prevent tumor immunity (6). However, the mechanisms of remote tumor cell-induced organ tolerance still require further clarification.

MDSCs, a heterogeneous of immune cells including immature DCs, macrophages, granulocytes, and other myeloid cells in early stages of their differentiation, usually express CD11b, CD33, and low levels of leukocyte antigen-DR in humans or CD11b and Gr1 in mice (7, 8). As reported by Gabrilovich and other scientists, as MDSC accumulated during advanced cancer stages, they exerted an immune-regulatory role and could inhibit many immune cells: CD4^+^, CD8^+^, NK, Tregs, etc. (3, 9, 10). Several mechanisms of MDSC suppressive functions have been described, including l-arginine depletion by the enzymes arginase 1 (Arg-1) or inducible nitric oxide synthase (iNOS) and generation of reactive oxygen species (ROS) (11–13). Moreover, MDSCs also secreted many immune-suppressive cytokines, such as IL-6, IL-10, and transforming growth factor β (TGF-β) (14). However, other mechanisms may have not been identified.

The liver is a blood-enriched organ and contains abundant innate and adaptive immune cell subtypes. MDSCs in hepatocellular carcinoma patients regulate the innate system and contribute to immune suppressor networks (15). However, liver tolerance mechanisms induced by remote tumor inoculated subcutaneously (s.c.) outside the liver are uncertain.

A T cell-dependent experimental hepatitis in mice induced by concanavalin A (Con A) was reported in 1990 by Tiegs (16). Con A-induced acute hepatitis is well documented and imitates human autoimmune diseases. IFN-γ plays a critical role in T cell-dependent liver injury in mice initiated by Con A (17, 18). In our previous studies, IFN-γ is critical for tumor immunity and γδ T cells provide the early source of IFN-γ (19). In the model of Con A-induced hepatitis, NK or NKT cells detrimented the liver damage trough making IFN-γ, which was negatively regulated by γδT cells (20). In this study, we intend to investigate the immune tolerance in the liver of tumor-bearing (TB) mice using Con A-induced hepatitis as the readout of liver immune response.

The TGF-β has three isoforms in mammalian animals, including TGF-β1, TGF-β2, and TGF-β3 that exert diverse roles in controlling cell proliferation, differentiation, wound healing, immune systems, and some pathological processes, e.g., fibrosis and cancer (21, 22). TGF-β1 is most highly expressed by immune cells, and a malfunction in this signaling pathway resulted in tumorigenesis (23). Increased TGF-β production has been reported in both human cancer patients and animal models, which is usually considered as a negative prognostic indicator (24). Pathological forms of TGF-β signaling promote tumor evasion of immune surveillance, tumor growth, and metastasis. Decreased TGF-β signaling reduces formation of gastrointestinal tumors (25). Our previous study also showed that tumor-derived TGF-β contributed to both tumor growth and tumor immunity (26). TGF-β exists in two forms: membrane bounded and soluble (27). However, the role of tumor-derived TGF-β in MDSC recruitment regulation was unclear.

In this report, we investigated the mechanisms of liver immune suppression induced by remote tumor cells. We demonstrated that growing tumor cells triggered the influx of MDSCs into the liver, and these MDSCs then suppressed the function of NKT cells through their membrane-bound TGF-β.

## Materials and Methods

### Mice

C57BL/6J [wild-type (WT)] mice were purchased from the Vital River Laboratory Animal Technology Co., Ltd. (Beijing, China). All mice were kept in specific pathogen-free conditions in the animal facility at Nankai University (Tianjin, China) and used at 6–10 weeks of age. CXCR2^−/−^ mice (on the C57BL/6J background) were purchased from Jackson Laboratory (Bar Harbor, ME, USA) *via* Nanjing Medicine University. All animal procedures were approved by the Nankai University Experimental Animal Ethics Committee.

### Cell Lines

B16-F0 (B16), TGF-β sh RNA-transfected stable B16-F0 (shB16) (26), and EL-4 cells were cultured in DMEM (Hyclone, ThermoFisher, Beijing, China) with 10% FBS and were maintained in a humidified incubator containing 5% CO_2_ at 37°C.

### Mice Model

Fulminant hepatitis was induced by Con A (10 mg/kg body weight) injected *via* tail vein. Tissue H&E stain and serum alanine aminotransferase (ALT) levels were measured to evaluate the mice model. TB mice were prepared by injecting tumor cells s.c. at 1 × 10^6^ per mouse. Tumor sizes were measured every 2 days, and Con A was treated on 15 days posttumor inoculation.

### Reagents

Concanavalin A, catalase, and l-*N*^6^-(1-iminoethyl) lysine dihydrochloride (l-NIL) were purchased from Sigma (Sigma-Aldrich). *N*-hydroxy-nor-arginine (nor-NOHA) was purchased from Caymen Chemical (MI, USA). Recombinant mouse (rm) TGF-β was purchased from R&D Systems (Minneapolis, MN, USA). FITC-conjugated CD11b (clone M1/70), PE-conjugated CD11b (clone M1/70), APC-conjugated Gr-1 (clone RB68C5), FITC-conjugated CD3 (clone 145-2C11), APC-conjugated NK1.1 (clone PK136), and PE-conjugated IFN-γ (clone XMG1.2) were purchased from BD Biosciences (San Jose, CA, USA). Ionomycin, GolgiStop, and PMA were purchased from BD Biosciences (San Jose, CA, USA). Anti-TGF-β1 Ab (clone 1D11) was obtained from R&D Systems (Minneapolis, MN, USA). Anti-IL-6 mAb (clone MP5-20F3), Anti-IL6R mAb (clone 15A7), neutralizing anti-Gr-1 Ab (clone RB68C), and anti-CD25 Ab (clone PC-61.5.3) were purchased from Tianjin Sungene (Tianjin, China).

### Histology

For histological analysis, mice livers with hepatitis were fixed with 4% (w/v) paraformaldehyde and then embedded in paraffin. Sections (4 μm) that had been deparaffinized and rehydrated were stained with H&E.

### ELISA

Mouse IFN-γ (cat. 430805), TNF-α (cat. 430905), IL-12 (cat. 433605), IL-4 (cat. 431105), and IL-6 (cat. 431305) ELISA kits were purchased from BioLegend (San Diego, CA, USA), and ELISA was performed according to the manufacturer’s protocols.

### Quantitative RT-PCR (qRT-PCR)

Total RNA was extracted from tissues or cells with Trizol reagent from Life Technologies (Carlsbad, CA, USA). qRT-PCR analyses for the mRNA of CXCRs and CXCLs were performed by using PrimeScript RT-PCR kits (Takara, Dalian, China). The mRNA level of glyceraldehyde-3-phosphate dehydrogenase was used as an internal control.

The primer sequences used are listed in Table S1 in Supplementary Material.

### Isolation of Liver Mononuclear Cells (MNCs)

Liver MNCs were isolated and purified by the method of Faheina-Martins et al. (28), with some modifications. Briefly, homogenized liver cells were resuspended in 40% percoll (GE Healthcare), gently overlayed onto 70% percoll, and centrifuged for 30 min at 1,260 × *g*. Finally, purified MNCs were collected from the interface for further study.

### Intracellular Staining and Flow Cytometry

Surface staining was performed with the corresponding fluorescence-labeled surface Abs. For intracellular IFN-γ staining, liver MNCs were obtained from the liver of mice and stimulated with PMA (50 ng/ml) and ionomycin (1 µg/ml) in the presence of GolgiStop for 6 h. Cells were then fixed, permeabilized, and stained as previously described (29).

### Detection of IFN-γ

A total of 1 × 10^6^/mL WT hepatic NKT cells (>95% pure, Figure S2 in Supplementary Material) were cultured alone or with TB hepatic MDSCs (>95% pure, Figure S2 in Supplementary Material) at a1:1 ratio or with reagents (0.5 ng/ml TGF-β1 or 10 ng/ml anti-TGF-β1 Ab) for 6 h. Then, NKT cells were stimulated with 25 ng/ml PMA and 1 µg/ml ionomycin for 18 h. IFN-γ concentrations in the supernatants were determined by ELISA kit from BioLegend (San Diego, CA, USA).

### Detection of ROS

The levels of intracellular ROS were detected by a ROS assay kit (Beyotime Biotech, China) following the manufacturer’s protocol. Briefly, liver MNCs isolated from both WT and TB mice were washed twice with PBS and incubated with DCFH-DA (10 µmol/l) at 37°C for 40 min and then analyzed through flow cytometry.

### Adoptive Transfer or Depletion of MDSCs and Tregs

Purified MDSCs or Tregs were intravenously (i.v.) injected into WT mice (5 × 10^5^ per mouse). For MDSC depletion, 0.2 mg of anti-Gr-1 Ab (RB6-8C5) was intraperitoneally (i.p.) injected into TB mice. Twelve hours later, mice were challenged by Con A. For Treg depletion, 0.2 mg of anti-CD25 Ab (PC-61.5.3) was i.p. injected into TB mice. Forty-eight hours later, mice were challenged by Con A. The cell transfer and depletion efficiency were confirmed by flow cytometry.

### Statistics

Data are presented as mean + SEM. Statistical significance between two groups was evaluated using a two-tailed unpaired Student’s *t*-test. Statistically significant data are indicated by asterisks (*p < 0.05, **p < 0.01, and ***p < 0.001).

## Results

### TB Mice Showed Alleviated Con A-Induced Fulminant Hepatitis

**Figure 1:**
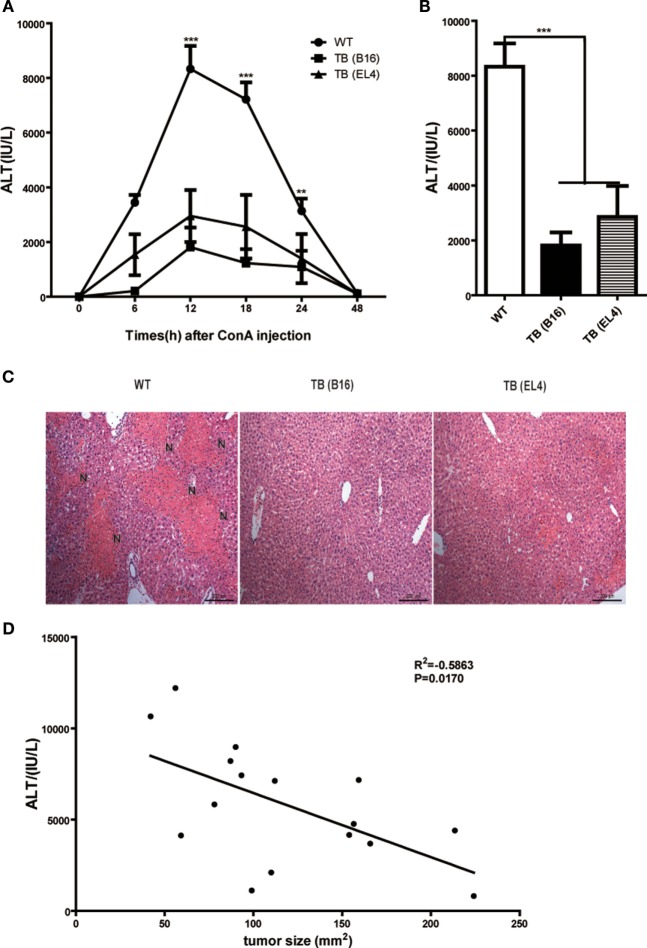
**Alleviated concanavalin A (Con A)-induced hepatitis in tumor-bearing (TB) mice**. Sex- and age-matched C57BL/6 wild-type mice were either untreated or subcutaneously injected with B16 or EL4 tumor cells (1 × 10^6^ cells/mouse) to prepare TB mice (TB-B16 or TB-EL4), respectively. On 15 days posttumor inoculations, these mice were challenged with Con A (10 mg/kg body weight). **(A)** Serum samples were collected at different time points post-Con A treatments and used for analysis of alanine aminotransferase (ALT) levels (*n* = 8). **(B)** Results from one representative experiment at the time point of 12 h posttreatment are shown. **(C)** Liver tissues collected at 12 h post-Con A treatments were fixed for hematoxylin and eosin staining, and one representative tissue staining is shown. Scale bars, 200 µm. **(D)** The relationship of serum ALT levels and tumor sizes was analyzed by SPSS, and results from one representative experiment are shown (*R*^2^ = −0.5863, *p* = 0.0170).

To study the role of growing living tumor cells in liver immune responses, C57BL/6J mice (WT mice) were inoculated s.c. either with 1 × 10^6^ B16 or EL4 cells to generate TB mice (TB-B16 or TB-EL4 mice), respectively, followed by Con A challenge (10 mg/kg body weight) 15 days posttumor inoculations. Serum ALT levels were measured at indicated time points (Figure 1A). Interestingly, TB mice were highly resistant to Con A and showed decreased serum ALT levels (Figure 1B) and attenuated liver damage (Figure 1C) when compared with WT mice. TB (B16) mice showed a more protective role in Con A-induced hepatitis; therefore, we used TB (B16) mice models in subsequent studies. Moreover, the relationship between ALT levels and tumor sizes were also analyzed. ALT levels were negatively correlated with tumor sizes (Figure 1D), but not with times of tumor growth (data not shown). These findings indicated that TB mice had a significant resistance against Con A-induced fulminant hepatitis.

### Reduced IFN-γ and Mediated Inflammation in TB (B16) Mice

**Figure 2:**
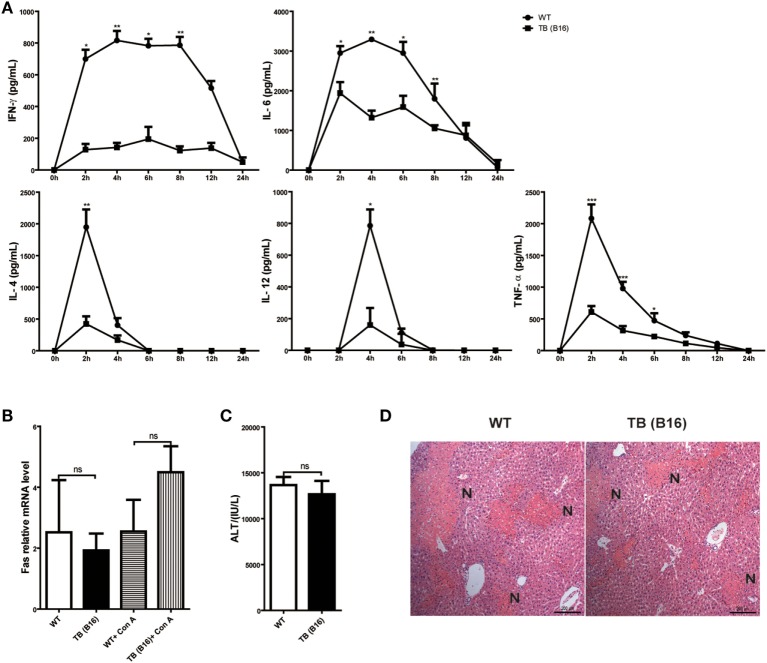
**Induction of immune suppression in tumor-bearing (TB) (B16) mice**. **(A)** Sex- and age-matched wild-type (WT) mice were either untreated or inoculated with B16 tumor cells as described above (named TB-B16), followed by concanavalin A (Con A) injections. Serum samples were collected at 0, 2, 4, 6, 8, 12, and 24 h post-Con A treatments and used for determining cytokine levels by ELISA (*n* = 6). Results shown are one of three independent experiments. **(B)** Livers of WT and TB mice were harvested before and 2 h after Con A challenge. Fas mRNA levels were determined *via* quantitative real-time PCR (*n* = 3). **(C)** A total of 1 mg/kg anti-Fas (clone Jo2) were injected intravenously into WT and TB mice. At 12 h after injections, serum alanine aminotransferase levels were measured (*n* = 6). **(D)** Liver tissues were fixed for hematoxylin and eosin staining, and one representative tissue staining is shown. Scale bars, 200 µm.

To define the mechanisms of liver immune suppression induced by remote tumor cells in TB (B16) mice, inflammatory cytokine levels of WT and TB (B16) mice were analyzed in sera samples collected at different time points post-Con A challenge (10 mg/kg body weight). A remarkable cytokine storm was observed after Con A challenge in WT mice (Figure 2A). Notably, compared with TB (B16) mice, an extremely high level of IFN-γ reached its peak in WT mice at 2 h and steadily persisted for 12 h. These findings suggested that IFN-γ exerted a critical pathogenic role in mediating Con A-induced liver injuries.

To exclude the possibility that the protective role of tumor burden was due to an intrinsic decreased susceptibility to apoptosis independent of inflammation, the response of hepatocytes to activating anti-Fas antibody was evaluated. Fas expression was not changed between WT and TB (B16) mice with or without Con A treatment (Figure 2B). Injection of this antibody induced hepatocyte apoptosis due to Fas-FasL-mediated death signaling. Results showed that WT and TB (B16) mice showed similar ATL levels (Figure 2C) and liver damage (Figure 2D) following this antibody treatment. Therefore, hepatocytes of TB (B16) mice had similar response to Fas-FasL-mediated apoptosis.

Taken together, these findings revealed that alleviated liver injury in TB (B16) mice is dependent on IFN-γ-mediated inflammation, but not due to Fas-FasL signaling.

### MDSCs, Not Tregs, Exerted Suppressive Function in Liver of TB (B16) Mice

**Figure 3:**
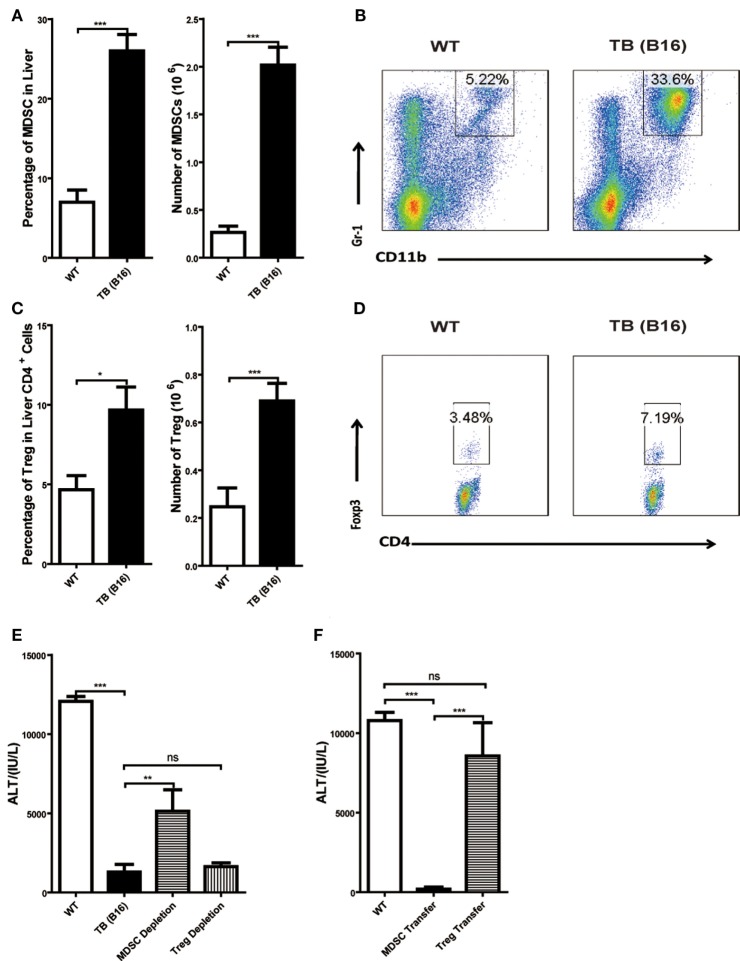
**Increased liver immune-suppressive cells in the liver of tumor-bearing (TB) mice**. Sex- and age-matched wild-type (WT) mice were either untreated or inoculated with 1 × 10^6^ B16 cells. Fifteen days postinoculation, liver mononuclear cells were prepared and analyzed through flow cytometry (*n* = 6). **(A)** The percentage and absolute number of CD11b^+^Gr1^+^ myeloid-derived suppressor cells (MDSCs) (mean ± SEM) are shown. **(B)** One representative FACS staining in panel **(A)** is shown. **(C)** The percentage and absolute number of CD4^+^Foxp3^+^ T regulatory cells (Tregs) (mean ± SEM) are shown. **(D)** One representative FACS staining in panel **(C)** is shown. **(E)** Depletion of MDSC, but not Tregs, restored concanavalin A (Con A)-induced hepatitis in TB mice. TB mice were either untreated or intraperitoneally injected with 0.2 mg anti-Gr1 (clone RB68C5) or anti-CD25 (clone PC-61.5.3) prior to Con A treatment as described in Section “Materials and Methods.” Serum samples collected at 12 h post-Con A treatment were used for alanine aminotransferase (ALT) detection, and results from one typical experiment are shown (mean ± SEM, *n* = 7). **(F)** Adoptive transfer of MDSCs, but not Tregs, from TB mice protected WT mice against Con A-induced hepatitis. Sex- and age-matched WT mice were either untreated or adoptively transferred with 5 × 10^5^ CD11b^+^Gr1^+^ cells or CD4^+^CD25^+^ Treg cells from TB mice *via* intravenous injection prior to Con A injection. Serum samples collected at 12 h post-Con A treatment were used for analyzing ALT levels. Results from one representative experiment are shown (mean ± SEM, *n* = 5). Data represent at least three independent experiments with similar results.

To further elucidate the liver immune environment in TB (B16) mice, liver MNCs were isolated from either WT or TB (B16) mice, and immune-suppressive cells, MDSCs, and Tregs were analyzed through flow cytometry. Both percentages and numbers of MDSCs and Tregs in TB (B16) mice were remarkably higher than in WT mice (Figures 3A,C). Meanwhile, one representative FACS staining showed that MDSCs (Figure 3B) and Tregs (Figure 3D) were accumulated in TB (B16) mice livers. Therefore, we assumed that alleviated inflammation and liver injuries in TB (B16) mice were due to hepatic MDSCs and/or Tregs accumulation.

To further elucidate the underlying mechanisms of immune-suppressive cells in protecting TB mice from liver injury, depletion and adoptively transfer experiments were performed. To deplete Tregs, anti-CD25 antibody (200 μg/mouse) was injected 48 h (Figure S1C in Supplementary Material) prior to Con A injection and serum ALT levels were detected 12 h post-Con A injection. To deplete MDSCs, TB (B16) mice were treated with anti-Gr-1 antibody (200 μg/mouse) 24 h (Figure S1A in Supplementary Material) prior to Con A injection and serum ALT levels were detected 12 h post-Con A injection. We demonstrated that it could restore ALT level only by depleting MDSCs, but not Tregs (Figure 3E). To further identify MDSC immune-suppressive function, either 5 × 10^5^ MDSCs or Tregs were isolated from the livers of TB (B16) mice and i.v. transferred into WT mice. MDSCs were recruited into livers 3 h (Figure S1B in Supplementary Material) posttransfer and Tregs at 6 h (Figure S1D in Supplementary Material) posttransfer. Thus, we injected Con A at these time points, respectively. Serum samples were collected 12 h post-Con A injection, and ALT levels were detected and found that adoptively transferred TB (B16) mice hepatic MDSCs, not Tregs, could protect against Con A-induced hepatitis (Figure 3F). These results further supported a critical role of MDSCs in tumor cell-induced liver immune suppression.

### IFN-γ Production of NKT Cells, but Not CD4^+^ T Cells, Was Inhibited by MDSCs

**Figure 4:**
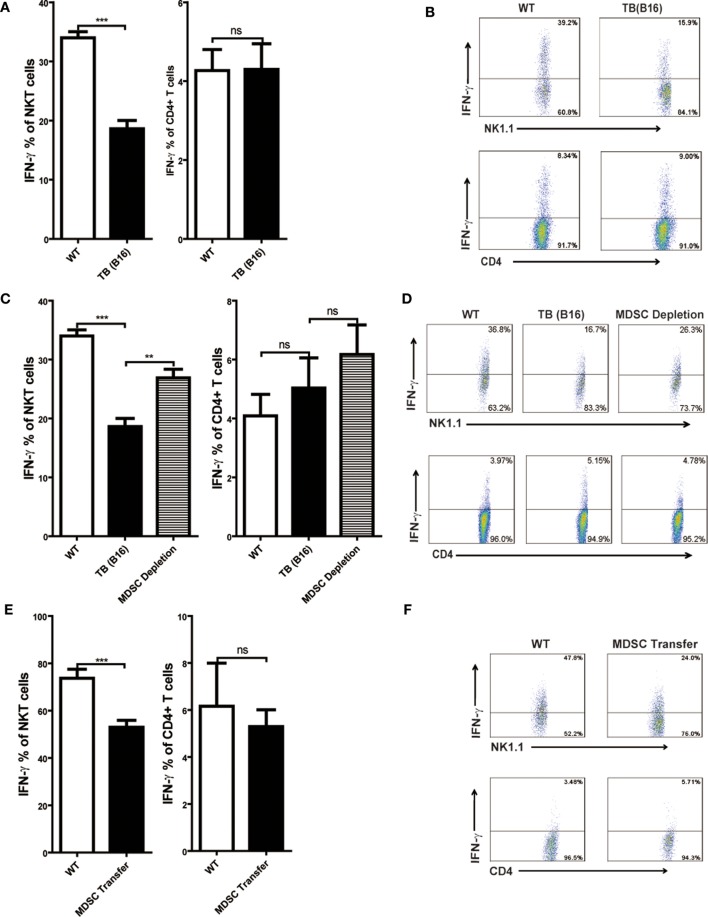
**IFN-γ production of NKT, not CD4+ T cells, was inhibited by myeloid-derived suppressor cells (MDSCs)**. **(A)** Wild-type (WT) and tumor-bearing (TB) mice were challenged by concanavalin A (Con A) (10 mg/kg body weight) 15 days post-B16 injections; 12 h later, mice were sacrificed and liver mononuclear cells were isolated. Afterward, intracellular IFN-γ intracellular staining was performed as described in Section “Materials and Methods,” and cells were analyzed by flow cytometry (*n* = 5). **(B)** One typical flow cytometry plot in panel **(A)** is shown. **(C)** MDSC depletion restored IFN-γ production of NKT cells. WT and TB mice were challenged by Con A 24 h after anti-Gr1 treatment. Then, 12 h later, mice were sacrificed and IFN-γ intracellular staining was performed, and cells were analyzed by flow cytometry (*n* = 5). **(D)** One typical flow cytometry plot in panel **(C)** is shown. **(E)** MDSC transfer inhibited IFN-γ production of NKT cells. 5 × 10^5^ TB MDSCs were intravenously transferred into WT mice, followed by Con A challenge (10 mg/kg), and 12 h later, livers were isolated. IFN-γ intracellular staining was performed, and cells were analyzed by flow cytometry (*n* = 5). **(F)** One typical flow cytometry plot in panel **(E)** is shown.

Consistent with other reports, Con A-induced hepatitis is related to IFN-γ production in the liver (18, 30). To elucidate the cellular source of IFN-γ, livers of WT and TB (B16) mice were isolated 12 h post-Con A injection, and IFN-γ production of NKT and CD4^+^ T cells was analyzed through flow cytometry. IFN-γ production of NKT, not CD4^+^ T cells, were inhibited in the liver of TB (B16) mice challenged by Con A (Figure 4A). One typical flow cytometry plot is shown (Figure 4B). To further identify whether the inhibition of IFN-γ production of NKT cells was due to accumulated MDSCs in livers of TB (B16) mice, we depleted MDSCs of TB (B16) mice by anti-Gr-1 antibody or transferred 5 × 10^5^ TB (B16) hepatic MDSCs into WT mice followed by Con A injection at indicated times. Livers were isolated 12 h post-Con A challenge and stimulated with 50 ng/ml PMA and 1 µg/ml ionomycin in the presence of GolgiStop for 6 h, and IFN-γ production of CD4^+^ and NKT cells was analyzed by flow cytometry. We found that IFN-γ production of NKT cells was restored after MDSC depletion (Figure 4C), as representative FACS plot is shown (Figure 4D). Interestingly, IFN-γ production of CD4^+^ T cells was not changed (Figures 4C,D). Similarly, adoptive transferring MDSCs also suppressed IFN-γ production only by NKT cells, not by CD4^+^ T cells (Figures 4E,F). Our results thus revealed that IFN-γ production of NKT, not CD4^+^ T cells, was inhibited by MDSCs accumulated in the liver of TB (B16) mice.

### MDSCs Inhibited IFN-γ Production of NKT Cells through Membrane-Bound TGF-β

**Figure 5:**
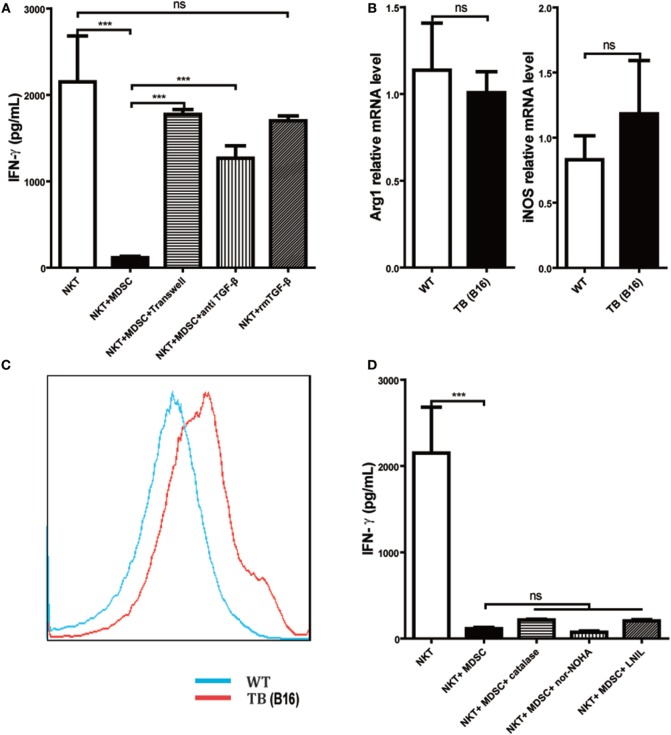
**Myeloid-derived suppressor cells (MDSCs) inhibited IFN-γ production of NKT cells *via* membrane-bounded transforming growth factor β (TGF-β) in a cell contact-dependent manner**. **(A)** Membrane-bound TGF-β is the main factor for suppression of NKT cell IFN-γ secretion. NKT cells (CD3^+^NK1.1^+^) were isolated from liver tissues of wild-type (WT) mice, co-cultured with MDSCs sorted from livers of tumor-bearing (TB) mice at the ratio of 1:1 under various conditions, including either transwell (0.4 µm) separation or in the presence of 10 ng/ml anti-TGF-β1 mAb or 0.5 ng/ml rmTGF-β1 for 6 h, and then cells were then stimulated with PMA (25 ng/ml) plus ionomycin (1 µg/ml). Eighteen hours later, supernatants were harvested for determination of IFN-γ levels using ELISA kit. Results (mean ± SEM) of triplicated wells from one representative experiment are shown. **(B)** Expression levels of arginase 1 (Arg-1) and inducible nitric oxide synthase (iNOS) were not changed in liver tissues of TB mice. Livers were isolated from either WT or TB mice (day 15 posttumor inoculation), and mRNA levels of Arg-1 and iNOS were determined *via* quantitative real-time PCR (*n* = 3). Results from one representative experiment are shown. **(C)** Reactive oxygen species (ROS) expression was increased in the liver of TB mice. Livers were isolated from either WT or TB mice 15 days posttumor inoculation. ROS levels were determined *via* ROS assay kit as described in Section “Materials and Methods” (*n* = 3). Results from one representative experiment are shown. **(D)** Arg-1, iNOS, and ROS inhibitors failed to restore IFN production of NKT cells *in vitro*. NKT cells were co-cultured with MDSCs isolated from the liver of TB mice at the ratio of 1:1 as described above in the absence or presence of various inhibitors, including *N*-hydroxy-nor-arginine (1 mM), catalase (1,000 U/ml), or l-NIL (0.5 µM) for 6 h. Then cells were stimulated with PMA plus ionomycin, and IFN-γ levels at the supernatant were determined using ELISA kit. Results from triplicated wells (mean ± SEM) are shown.

To further investigate the underlying mechanisms for impairment of NKT cell function by hepatic MDSCs in TB (B16) mice, we co-cultured WT hepatic NKT with MDSCs sorted from livers of TB (B16) mice at a 1:1 ratio or at various conditions for 6 h and then stimulated with 25 ng/ml PMA plus 1 µg/ml ionomycin for 18 h. It showed that co-culturing with TB (B16) MDSCs inhibited IFN-γ production of NKT cells (Figure 5A). To identify whether soluble molecules or cell–cell interactions were involved in this process, we incubated NKT cells and MDSCs in the transwell system (0.4 µM). The downregulation of IFN-γ production of NKT cells by MDSCs was lost in the transwell system (Figure 5A), suggesting that MDSC-mediated inhibition of NKT cells was due to cell–cell contact. As TGF-β1 is an important suppressive cytokine produced by MDSCs (31), we investigated whether MDSCs could express membrane-bound TGF-β1, and if so, whether membrane-bound TGF-β1 was involved in the process. Neutralization of TGF-β1 in the co-culture system restored IFN-γ production (Figure 5A), whereas the exogenous recombinant TGF-β1 supplement in the culture system had no significant suppressive effect. Meanwhile, we also detected other MDSC-derived suppressive factors, Arg-1, iNOS, and ROS. Arg-1 and iNOS expression were not changed between WT and TB (B16) mice (Figure 5B); however, ROS expression elevated in TB (B16) mice livers (Figure 5C). To verify whether Arg-1, iNOS, or ROS participated in the inhibition of NKT by MDSCs, we co-cultured WT NKT with MDSCs sorted from livers of TB (B16) mice at a 1:1 ratio in the absence or presence of specific inhibitors of Arg-1, ROS, or iNOS: nor-NOHA (1 mM), catalase (1,000 U/ml), or l-NIL (0.5 µM), respectively (32). We confirmed that Arg-1, iNOS, and ROS produced by MDSCs are not involved in suppressing IFN-γ production; this is because inhibitors against these suppressive factors could not restore the suppression of NKT cells by MDSCs in the co-culture system (Figure 5D). Therefore, we concluded that the membrane-bound TGF-β1 from MDSCs was responsible for the impairment of IFN-γ production of NKT cells.

### MDSCs Recruited into Liver *via* CXCR2 Signal Pathway

**Figure 6:**
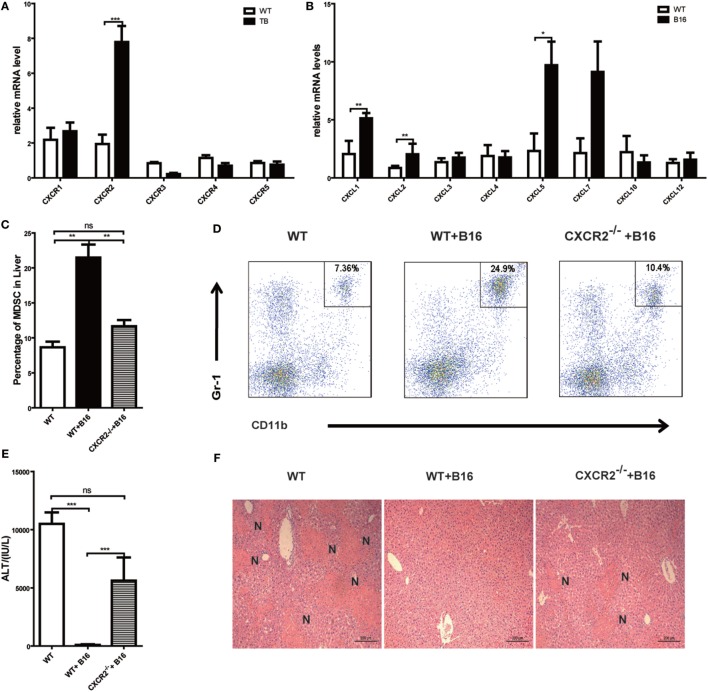
**Recruitment of myeloid-derived suppressor cells (MDSCs) into tumor-bearing (TB) (B16) mice livers was CXCR2 mediated**. **(A)** Liver mononuclear cells (MNCs) were isolated from either wild-type (WT) or TB mice 15 days posttumor inoculation, and mRNA levels of CXCRs were determined *via* quantitative real-time PCR (*n* = 3). **(B)** Liver tissues were isolated from either WT or TB mice 15 days posttumor inoculation, and mRNA levels of CXCRs were determined *via* quantitative real-time PCR (*n* = 3). **(C)** CXCR2-dependent liver recruitment of MDSC in TB mice. Sex- and age-matched WT mice and CXCR2^−/−^ mice were either untreated or inoculated with B16 tumor cells to prepare TB mice as described above, and liver MNCs was prepared from the livers and then used for analyzing the percentage of CD11b^+^Gr1^+^ through flow cytometry (*n* = 6). **(D)** One representative FACS plot in panel **(C)** is shown. **(E)** Deficiency of CXCR2 rendered susceptibility to Con A-induced liver damage in TB mice. Sex- and age-matched WT or CXCR2^−/−^ mice were injected with B16 tumor cells, and on day 15 posttumor inoculation, mice were challenged with concanavalin A (Con A). Serum samples collected at 12 h post-Con A injections were used for analyzing the levels of alanine aminotransferase (*n* = 6). **(F)** Liver tissues were fixed for hematoxylin and eosin staining, and one representative tissue staining is shown. Scale bars, 200 µm.

Results above revealed that MDSCs were critical for liver immune suppression in TB (B16) mice. CXCR-CXCL signal pathways have been reported to exert an important role in MDSC migration (33, 34). To further define the mechanisms of MDSC recruitment into liver, CXCRs and CXCLs expressions in liver lymphocytes was analyzed *via* quantitative real-time PCR. Interestingly, CXCR2 expression was increased in TB (B16) mice (Figure 6A). Meanwhile, CXCR2 ligands, CXCL1, CXCL2, and CXCL5 expressions were also elevated in TB (B16) mice livers (Figure 6B). These findings suggested a hypothesis that MDSCs recruited into livers in TB (B16) mice *via* CXCR2 signaling. To test this hypothesis, CXCR2^−/−^ mice were inoculated with B16 tumor cells. Interestingly, recruitment of MDSCs into the liver was significantly impaired in CXCR2^−/−^ TB (B16) mice (Figure 6C), and one example of representative FACS plot is shown (Figure 6D). Moreover, WT-TB and CXCR2^−/−^ TB were challenged by Con A 15 days posttumor inoculations and found that CXCR2^−/−^ TB (B16) mice partly restored sera ALT levels and liver damage (Figures 6E,F). Collectively, these results demonstrated that CXCL1/2/5-CXCR2 signaling is responsible for recruitment of MDSCs into the liver.

### Tumor-Derived TGF-β-Regulated MDSC Recruitment into Liver *via* Regulating CXCLs Expression

**Figure 7:**
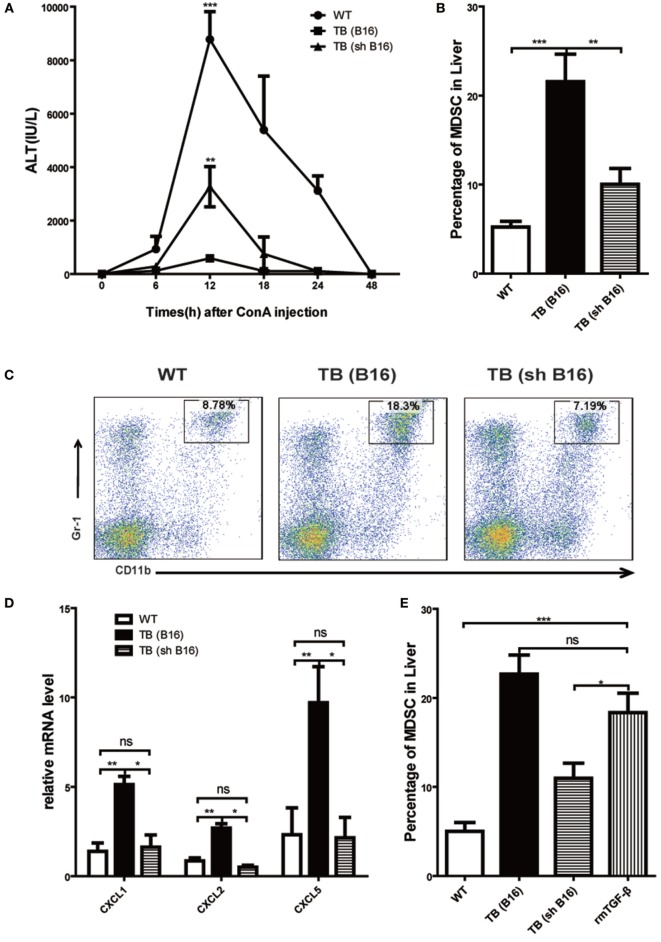

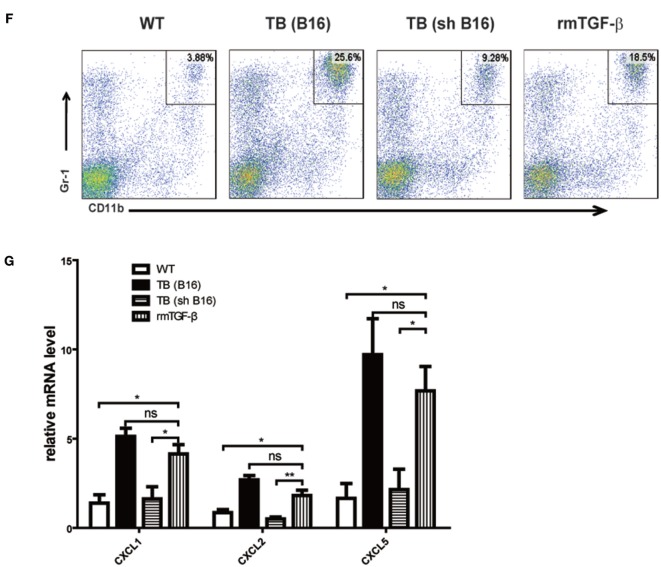
**Recruitment of myeloid-derived suppressor cells (MDSCs) into livers was regulated by tumor-derived transforming growth factor β (TGF-β)**. **(A)** Tumor-derived TGF-β contributed to tumor-induced immune suppression in livers. Sex- and age-matched WT mice were either untreated or inoculated with B16 cells or sh RNA-transfected stable B16 cells prepared in our laboratory (1 × 10^6^ cells/mouse) and named TB-B16 or TB-sh B16, respectively. After 15 days posttumor inoculations, mice were challenged with concanavalin A; 12 h later, serum alanine aminotransferase levels were analyzed. Results from one of three independent experiments are shown (*n* = 8). **(B)** Recruitment of MDSCs into livers was dependent on tumor-derived TGF-β. Three groups of mice, as described above after day 15 posttumor inoculations, were used for percentage analysis of CD11b^+^Gr1^+^ MDSCs through flow cytometry (*n* = 6). **(C)** One representative FACS plot in panel **(B)** is shown. **(D)** Tumor-derived TGF-β elevated expression of CXCLs in the liver. Liver tissues collected from the three groups of mice as shown above were used for analyzing mRNA levels of CXCLs *via* quantitative real-time PCR (*n* = 3). **(E)** WT mice were intraperitoneally injected 1 µg rmTGF-β 3 h prior to sacrifice. Then WT, TB (B16), TB (sh B16), and WT injected rmTGF-β were sacrificed for analyzing the percentage of CD11b^+^Gr1^+^ MDSCs through flow cytometry (*n* = 3). **(F)** One representative FACS plot in panel **(E)** is shown. **(G)** Liver tissues collected from the four groups of mice in panel **(E)** were used for analyzing mRNA levels of CXCLs *via* quantitative real-time PCR (*n* = 3).

Our previous research disclosed that tumor-derived TGF-β induced MDSCs migration into tumor sites (26). To investigate tumor-derived TGF-β effects in MDSC recruitment into liver and immune suppression conduction, sex- and age-matched WT mice were either untreated or inoculated with B16 cells or shRNA-transfected stable B16 cells prepared in our laboratory (26) (Figure S3A in Supplementary Material) (1 × 10^6^ cells/mouse), which named TB (B16) or TB (sh B16), respectively (Figure S3B in Supplementary Material). On day 15 posttumor inoculations, these mice were challenged with Con A and levels of ALT in sera at indicated time points ware analyzed. It was found that TB (sh B16) mice could restore sera ALT levels (Figure 7A). Moreover, TB (sh B16) mice showed a lower percentage of hepatic MDSCs than TB (B16) mice (Figure 7B). One typical staining is shown (Figure 7C). Therefore, we assumed that tumor-derived TGF-β regulated MDSC recruitment into their livers. To further investigate the underlying mechanisms of MDSC recruitment, CXCR2 ligands, CXCL1, CXCL2, and CXCL5 expressions in liver tissues of WT, TB (B16), and TB (sh B16) mice was analyzed *via* quantitative real-time PCR. CXCL1, CXCL2, and CXCL5 expressions in TB (B16) mice were remarkably higher than in TB (sh B16) mice (Figure 7D). These findings suggested a hypothesis that tumor-derived TGF-β upregulated CXCL1, CXCL2, and CXCL5 expressions and then regulated MDSC recruitment into the liver. To further define tumor-derived TGF-β-regulated recruitment of MDSCs into the liver, we injected i.p. 1 μg/mouse rmTGF-β into WT mice. We found that injection of rmTGF-β promoted recruitment of MDSC into the liver (Figures 7E,F). To further analyze mechanism-mediated MDSCs recruitment, CXCR2 ligands expression was determined using quantitative real-time PCR and found that i.p. injection of rmTGF-β remarkably promoted CXCL1, CXCL2, and CXCL5 expressions in the liver (Figure 7G). These results revealed that tumor-derived TGF-β-regulated MDSCs recruitment into the liver *via* promoting CXCL1, CXCL2, and CXCL5 expressions in livers.

## Discussion

One of the biggest challenges for curing cancer is to fully understand the molecular mechanisms of how tumor cells escape from immune surveillance. It has been well documented that growing tumor cells release suppressing factors, which in turn creates special microenvironment that not only promotes tumor growth but also blocks immune clearance (35, 36). However, the underlying mechanisms concerning remote growing tumor-inducing organ-specific immune suppression are completely elusive. In this study, we demonstrated that remote growing tumor cells induced liver immune suppression through enhancing both the recruitment of MDSCs into liver tissues and their suppressive function on NKT cells.

One of the critical findings from our research determined the important role of MDSCs in inducing liver immune suppression. It has been well documented that upon subcutaneous tumor inoculation, large amounts of MDSCs flow into the liver (37). We demonstrated that increased numbers of MDSCs were responsible for immune suppression against Con A-induced hepatitis, confirmed by observations that depletion of these cells by anti-Gr1 antibodies restored Con A-induced liver inflammation, whereas adoptive transferring MDSCs from TB mice restrained Con A-triggered liver injury. It has to be emphasized that although the numbers of CD4^+^ Foxp3^+^ Tregs were also increased significantly in TB mice in comparison to those in WT mice, neither depletion nor adoptive transferring of Tregs was able to alter Con A-induced hepatitis, supporting our conjecture that accumulated MDSCs suppressed liver immune responses. These Tregs might be recruited or locally expanded with the factors secreted by MDSCs. It would be interesting to examine the interaction between these two sets of immune-suppressive cells for a more desirable understanding of the molecular mechanisms of tumor-mediated immune suppression.

Myeloid-derived suppressor cells have broad suppressive functions on many cell types. However, the underlying mechanisms for these cells to trigger liver immune suppression have not been elucidated. Con A-induced liver injury is a well-studied model, and both CD4^+^ T cells and NKT cells play important roles in mediating liver inflammation through producing IFN-γ. Our next important finding was clarifying the purpose of NKT cells, not CD4^+^ T cells, as the target of MDSCs in the liver. We observed that the numbers of IFN-γ-producing NKT cells were significantly reduced in TB mice in comparison with those in WT mice, and depletion of MDSCs restored the ability of NKT cells to produce IFN-γ. Consistently, adoptive transferring of MDSCs from TB mice inhibited IFN-γ production by NKT cells. In contrast, CD4^+^ T cells from all these experimental conditions showed no significant changes in their ability to produce IFN-γ. Therefore, we concluded that MDSCs recruited into the liver actively suppressed the function of NKT cells, resulting in liver immune suppression. Which functional molecules are essential for the suppression function of MDSCs on NKT cells? Importantly, we emphasized that interaction between MDSCs and NKT cells required cell contact, and the suppression effect was significantly blocked by anti-TGF-β antibodies. Interestingly, recombinant TGF-β1 failed to suppress the production of IFN-γ by NKT cells. Our concluding results were that membrane-bound TGF-β mediated the suppression function of MDSCs on NKT cells through cell contact-dependent mechanisms. These were consistent with a previous report that determined MDSCs induce NK cell anergy though membrane-bound TGF-β (10). The target of these membrane-bound TGF-β and downstream signaling pathways are unclear at the present time. It is also worth further investigating why the membrane-bound TGF-β has no effect on CD4^+^ T cells. Understanding the selectivity of each suppression molecules released from these negative regulators will lead to better designing therapeutics against these targets.

It has been observed and further confirmed that TB mice induced an influx of MDSCs into the liver. However, the underlying molecular mechanisms for this process are not fully understood. We determined that tumor-derived TGF-β played an important role in this influx, evidenced by the fact that inoculation of shRNA-TGF-β transfected stable cell line previously prepared (26) significantly reduced the accumulation of MDSCs into the liver. Furthermore, we elucidated that knockdown TGF-β from tumor cells significantly reduced the expression of chemokines (CXCL1, 2, and 5) in liver tissues, resulting in much less influx of MDSCs into the liver. This hypothesis was further confirmed by the fact that inoculation of B16 tumor cells into the CXCR2^−/−^ mice significantly blocked the recruitment of MDSCs into the liver. In summary, our results established a critical role of tumor-derived TGF-β in triggering the influx of MDSCs into the liver through facilitating the interaction between the CXCL1/2/5 and the CXCR2.

Interestingly, there was a report that MDSCs in TB mice promoted Con A-induced hepatitis (38), although the report does not clarify tumor sizes and days of posttumor inoculations when Con A was administered. How is the discrepancy explained between our results and those in the report? One possible explanation is the different intestinal microbiota, resulting from various mice sources and facilities in which they were hosted (39, 40). This is not the first time that phenotype differ from each other, when hosted in different environments. For example, our laboratory showed a much worse Con A-induced hepatitis in IL-17A^−/−^ mice, which differed dramatically from the results published in the US laboratories (20, 41). We cannot exclude other possibilities that tumor cells were used and their tumor sizes when Con A was injected.

In summary, we defined a critical role of MDSCs in inducing liver immune suppression in the TB hosts and emphasized the significance of targeting these cells in restoring antitumor immunity.

## Ethics Statement

This study was carried out in accordance with the recommendations of “Nankai University Experimental Animal Ethics Committee.” The protocol was approved by the “Nankai University Experimental Animal Ethics Committee.”

## Author Contributions

Conception and design: ZYin, LZ, ZW, HZhou, and HZhang. Development of methodology: HZhang and LW. Acquisition of data: HZhang, LW, ZL, GT, JT, and ZYang. Analysis and interpretation of data: HZhang and LW. Writing, review, and/or revision of the manuscript: HZhang, GC, and ZYin. Administrative, technical, or material support: ZYin. Study supervision: ZYin, LZ, and ZW.

## Conflict of Interest Statement

The authors declare that the research was conducted in the absence of any commercial or financial relationships that could be construed as a potential conflict of interest.

## Abbreviations

Con A, concanavalin A; MDSCs, myeloid-derived suppressor cells; TGF-β, transforming growth factor β; CXCR, CXC chemokine receptor; CXCL, CXC chemokine ligand; WT, wild type; TB, tumor bearing.
