# Supplementary material for: Critical Role of Myeloid-Derived Suppressor Cells in Tumor-Induced Liver Immune Suppression through Inhibition of NKT Cell Function
Source: Front Immunol. 2017 Feb 13;8:129. doi: 10.3389/fimmu.2017.00129 (PMC5303828; doi:10.3389/fimmu.2017.00129)
Supplement: Supplementary file 1 [file Data_Sheet_1.PDF]

Supplementary Table 1: the primer sequences used in this paper.

**Fas-F**	5’- TGG CAA GTT GAA GGG TG-3’
**Fas-R**	5’- GGC GGG TAG CAA ATG A-3’
**ARG-1-F**	5’-CTCCAAGCCAAAGTCCTTAGAG-3’
**ARG-1-R**	5’-AGGAGCTGTCATTAGGGACATC-3’
**iNOS-F**	5’-GTTCTCAGCCCAACAATACAAGA-3’
**iNOS-R**	5’-GTGGACGGGTCGATGTCAC-3’
**CXCR1-F**	5’-TCTGGACTAATCCTGAGGGTG-3’
**CXCR1-R**	5’-GCCTGTTGGTTATTGGAACTCTC-3’
**CXCR2-F**	5’-ATGCCCTCTATTCTGCCAGAT-3’
**CXCR2-R**	5’-GTGCTCCGGTTGTATAAGATGAC-3’
**CXCR3-F**	5’-TACCTTGAGGTTAGTGAACGTCA-3’
**CXCR3-R**	5’-CGCTCTCGTTTTCCCCATAATC-3’
**CXCR4-F**	5’-GACTGGCATAGTCGGCAATG-3’
**CXCR4-R**	5’-AGAAGGGGAGTGTGATGACAAA-3’
**CXCR5-F**	5’-ATGAACTACCCACTAACCCTGG-3’
**CXCR5-R**	5’-TGTAGGGGAATCTCCGTGCT-3’
**CXCL1-F**	5’-GGCTTCCTTATGTTCAAACAGGG-3’
**CXCL1-R**	5’-GCCGTTACTCGGGTAAATTACA-3’
**CXCL2-F**	5’-CCAACCACCAGGCTACAGG-3’
**CXCL2-R**	5’-GCGTCACACTCAAGCTCTG-3’
**CXCL3-F**	5’-CCATCCAGAGCTTGACGGTGAC-3’
**CXCL3-R**	5’-GGCTCAGCTGGACTTGCCGCTC-3’
**CXCL4-F**	5’-CCTGTATCCTGGGTTTCCGGACTGG-3’
**CXCL4-R**	5’-AAGTTCTACCTTGAGGAAATGG-3’
**CXCL5-F**	5’-GCGTCACACTCAAGCTCTG-3’
**CXCL5-R**	5’-GCGGCTATGACTGAGGAAGG-3’
**CXCL7-F**	5’-AACATAGCAGCAGTGTTCTGGGCAG-3’
**CXCL7-R**	5’-CAACTTGGCTTGCCCGTCT-3’
**CXCL10-F**	5’-GGATCCCTCTCGCAAGGACGGTCCG-3’
**CXCL10-R**	5’-GATAGGCTCGCAGGGATGA-3’
**CXCL12-F**	5’-CCTTCAGATTGTTGCACGGC-3’

**CXCL12-R**	5’-CATCTCCCACGGATGTCAGC-3’
**GAPDH-F**	5’-AAC ATC ATC CCT GCA TCC ACTG-3’
**GAPDH-R**	5’-CCT GCT TCA CCA CCT TCT TGAT-3’

F: forward primer; R: reverse primer

Supplementary Figures and Legends

Supplementary Figure 1

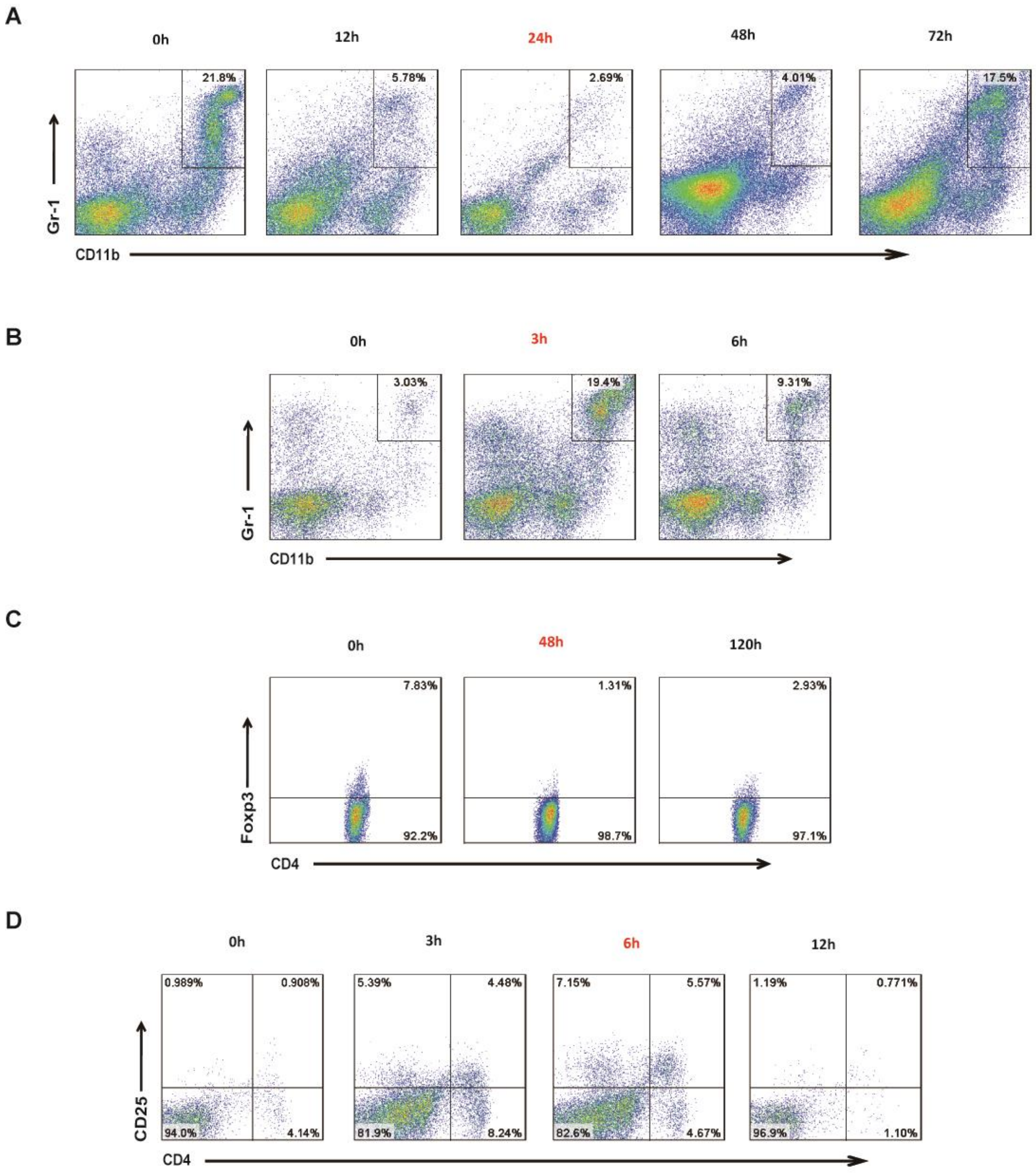

Supplementary Figure 1. Cell depletion and transfer efficiency. (**A**) For depletion of MDSCs, anti-Gr1 Ab was injected intraperitoneally (i.p.) into tumor-bearing (TB) mice and then sacrificed at different time points and MDSC percentage were calculated by flow cytometry. (**B**) MDSCs, sorted from TB livers, adoptively transferred inravenously (i.v.) into wild type (WT) mice, and then sacrificed at

different time points and MDSC percentage were calculated by flow cytometry. (**C**) For depletion of Tregs, anti-CD25 Ab was injected i.p. into TB mice and then sacrificed at different time points and Treg percentage were calculated by flow cytometry. (**D**) Tregs, sorted from TB livers, adoptively transferred into WT mice, and then sacrificed at different time points and Treg percentage were calculated by flow cytometry.

Supplementary Figure 2

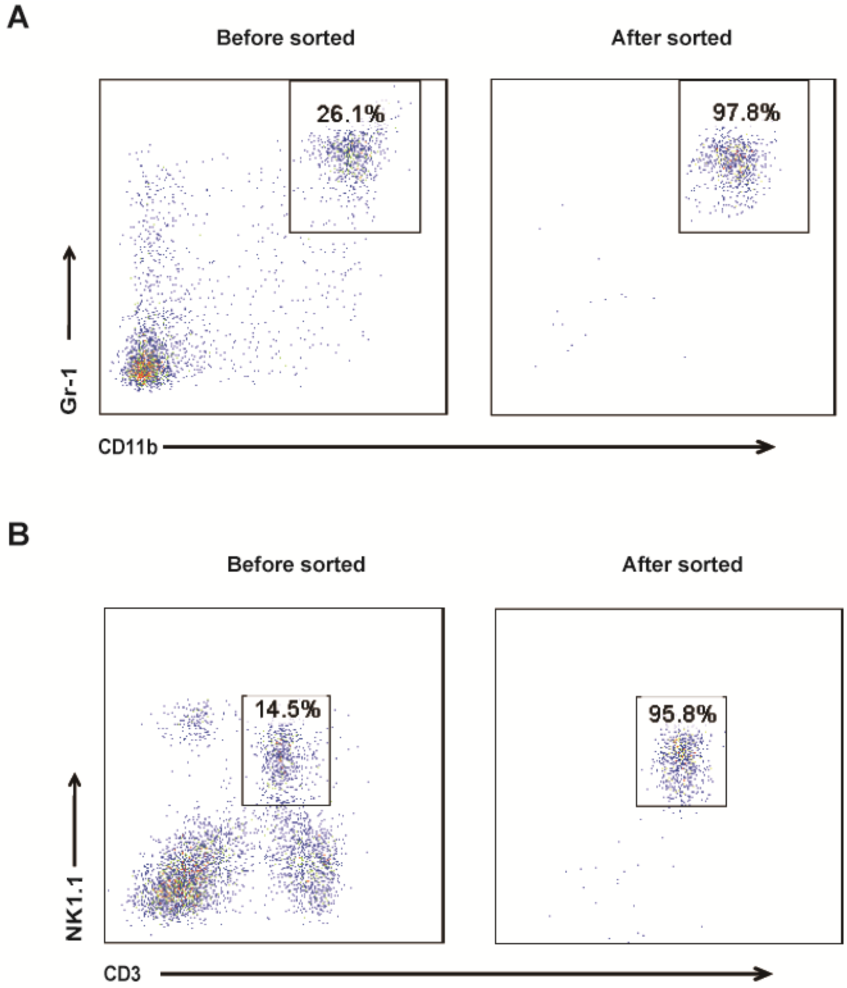

Supplementary Figure 2. Cells sorting efficiency. Liver MNC were isolated using

percoll, and MDSCs (**A**) and NKT cells (**B**) were sorted by Aria Ⅲ (BD) respectively (>95% pure).

Supplementary Figure 2

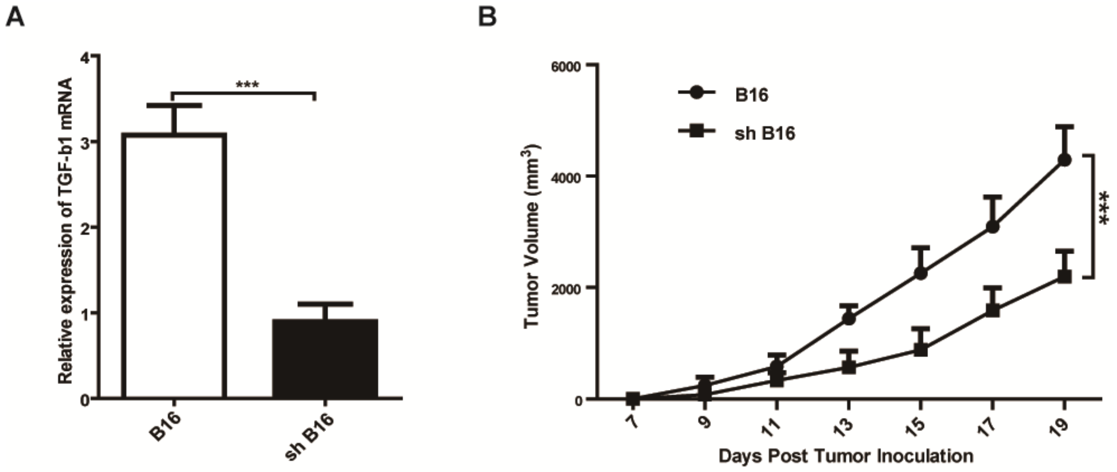

Supplementary Figure3. Tmor size of TB (B16 or sh B16) mice. (**A**) B16 and sh B16 cells were harvested and mRNA levels of TGF-β were analyzed via quantitative real-time PCR (n=3). (**B**) Sex and age matched C57BL/6 mice were either untreated or inoculated with B16 cells or sh B16 (1×10^6 cells /mouse) and tumor volumes were measured every two days.
